# Diagnostic accuracy of abbreviated biparametric MRI for prostate cancer screening: a prospective feasibility study (ReIMAGINE study)

**DOI:** 10.1007/s00330-025-11837-1

**Published:** 2025-08-06

**Authors:** Natasha Thorley, Tom Parry, Francesco Giganti, Douglas Kopcke, Harbir S. Sidhu, Giorgio Brembilla, Emma Stallard, Mark Emberton, Caroline M. Moore, Shonit Punwani

**Affiliations:** 1 Centre for Medical Imaging, University College London, London, UK; 2 Department of Radiology, University College London Hospital NHS Foundation Trust, London, UK; 3 Division of Surgical and Interventional Science, University College London, London, UK; 4 Department of Radiology, North Bristol NHS Trust, Bristol, UK; 5 Università Vita-Salute San Raffaele, Milano, Italy; 6 Department of Urology, University College London Hospitals NHS Foundation Trust, London, UK

**Keywords:** Prostate cancer, Screening, Magnetic resonance imaging, Prostate, Biparametric

## Abstract

**Objectives:**

To evaluate the diagnostic accuracy and imaging findings of an abbreviated biparametric MRI (bpMRI) protocol for prostate cancer (PCa) screening.

**Materials and methods:**

In this single-centre, prospective study, men aged 50–75 years were randomly selected for invitation to screening through participating general practices. Participants underwent screening with abbreviated bpMRI (axial T2-weighted and b2000 diffusion-weighted sequences) and PSA testing between October 2019 and December 2020. Screening MRIs were independently reported by two radiologists as either positive or negative by screening criteria, with a third radiologist if there was disagreement. Positive MRI or raised PSA density (PSAd) (≥ 0.12 ng/mL^2^) triggered standard-of-care NHS PCa assessment. Outcomes of the NHS assessment were collated as a composite reference standard and included multiparametric MRI ± biopsy, and a 2-year healthcare record follow-up. Diagnostic accuracy was evaluated using positive predictive value (PPV).

**Results:**

Among 303 men who completed screening (median age 62 years [IQR 56, 68]), 16% (48/303) had a positive screening MRI, and an additional 5% (16/303) had raised PSAd alone. Of 61 men referred to secondary care, 48% (29/61) had clinically significant PCa, with 2 (3%, 2/61) additional diagnoses during 2-year follow-up. Of 31 men with clinically significant PCa, 87% (27/31) had a positive screening MRI and 42% (13/31) had raised PSAd. The PPV of the screening MRI was 59% (27/46, 95% CI: 44, 72).

**Conclusion:**

Abbreviated bpMRI may have value in PCa screening independently of PSA testing. However, prospective multicentre evaluation is needed to assess the feasibility and cost-effectiveness of MRI-based screening at a national level.

**Key Points:**

***Question*** *MRI may reduce over- and under-diagnosis from prostate-specific antigen (PSA) testing in PCa screening, but its use is limited by long scan times, cost, and availability*.

***Findings*** *Abbreviated bpMRI identified more men with clinically significant cancer than PSAd (≥* *0.12* *ng/mL*^*2*^*), with a PPV of 59% (95% CI: 44, 72)*.

***Clinical relevance*** *Prostate MRI may improve cancer detection independently of PSA testing, with abbreviated protocols enhancing feasibility and scalability. While MRI may offer an opportunity for early diagnosis, further prospective, multicentre evaluation is needed to explore its potential role in screening settings*.

**Graphical Abstract:**

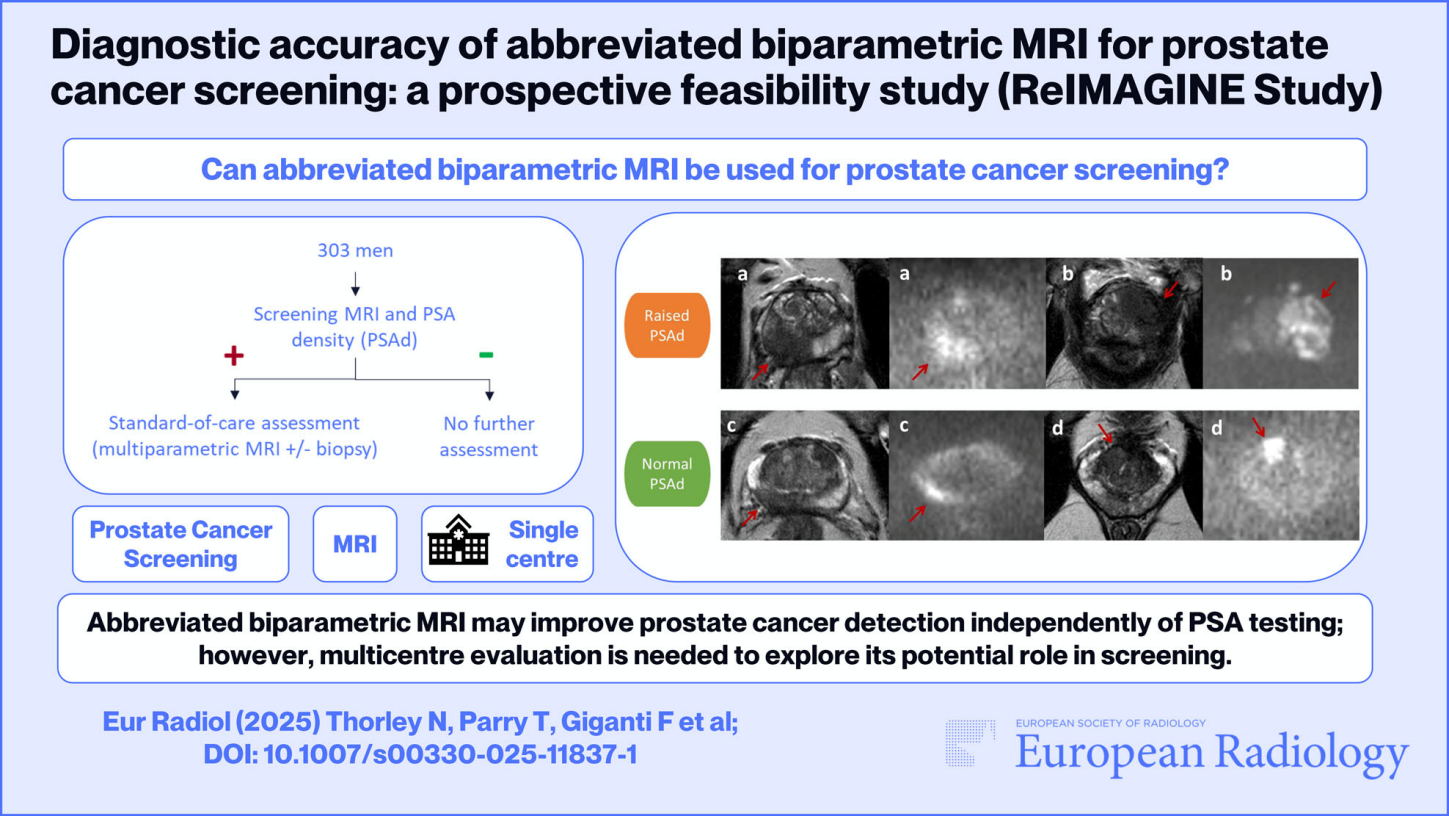

## Introduction

Prostate cancer (PCa) remains one of the leading causes of cancer-related deaths [1], and an effective population-based screening strategy has yet to be established. As the disease is often asymptomatic in the early stages, it presents a significant diagnostic challenge.

The European Randomised study of Screening for PCa demonstrated that population-based screening using prostate-specific antigen (PSA) testing reduces PCa mortality [2]. However, PSA has limited sensitivity and specificity, leading to both under- and overdiagnosis, with potential harms from unnecessary investigations, treatment, and follow-up [3, 4].

Multiparametric MRI (mpMRI) plays an established role in the diagnostic pathway [5–7], reducing unnecessary biopsies due to its high negative predictive value (NPV) for clinically significant prostate cancer (csPCa) and enabling targeted biopsies [8]. However, mpMRI is limited by long scan times, high cost, limited availability, and potential safety concerns regarding gadolinium-based contrast agents.

This has prompted interest in shorter, non-contrast protocols, such as biparametric MRI (bpMRI), which offer reduced scan times while maintaining similar diagnostic accuracy for csPCa [9–12]. Prospective screening studies, such as IP1-PROSTAGRAM [13], MVP [14], and PROSA [15], suggest that bpMRI may outperform PSA in detecting csPCa, supporting its potential role in population screening.

Even shorter, abbreviated bpMRI protocols could further improve the feasibility and scalability of MRI-based screening by reducing scan time and cost [10]. Although evidence on their diagnostic performance in screening populations is currently limited, studies in diagnostic settings have shown that abbreviated bpMRI achieves comparable accuracy to standard bpMRI and mpMRI [9, 10]. Notably, Brembilla et al found that an abbreviated bpMRI protocol using only axial T2-weighted imaging (T2WI) and high *b*-value (2000 s/mm²) diffusion-weighted imaging (DWI) had a higher positive predictive value (PPV) [9], possibly due to omission of apparent diffusion coefficient (ADC) maps. In a screening context, a higher PPV could help to balance the benefits of detecting csPCa against the harms of false positives and unnecessary interventions.

In the ReIMAGINE Prostate Cancer Screening study [16, 17], we utilised a two-sequence abbreviated bpMRI protocol and a novel binary reporting system designed to apply a high threshold for screen positivity. Here, we describe the imaging findings and evaluate the diagnostic accuracy of this simplified MRI-based screening approach.

## Materials and methods

### Study design

Ethical approval was granted by the London—Stanmore Research Ethics Committee Health Research Authority (ref19:/LO/1129), and study participants provided written informed consent. The ReIMAGINE Prostate Cancer Screening study was a prospective, population-based single-site study to assess the feasibility of MRI as a screening tool for PCa carried out at University College London Hospital National Health Service (NHS) Foundation Trust (ClinicalTrials.gov: NCT04063566). The study protocol [16] and primary outcomes [17] have been previously published, with this paper providing an analysis of the diagnostic accuracy.

### Participants

Potential participants were identified by screening patient databases at eight London primary care practices, and eligible men were randomly selected for invitation to screening via letter. Inclusion criteria included men aged 50–75 years with no prior PCa diagnosis or treatment. In the absence of established guidelines for screening age, the age range was selected based on patient and public involvement during protocol development. Men with contraindication to MRI or unable to provide informed consent were excluded. Interested men attended a screening assessment between October 2019 and December 2020.

### Procedures

#### Screening pathway

Participants underwent screening with a PSA blood test and a screening MRI scan.

The screening MRI was performed on a single 3-T scanner (Achieva, Philips Healthcare) using a pelvic phased array coil. The abbreviated bpMRI protocol included two sequences: small field-of-view axial T2WI (slice thickness: 3 mm; TR/TE: 5407/100 ms) and high *b-*value (2000 s/mm²) DWI (slice thickness: 5 mm; TR/TE: 2000/78 ms), with total acquisition time of < 10 min. Isotropic DWI images were derived from acquisitions in three orthogonal directions. Intravenous contrast agents were not administered, and ADC maps were not derived.

The screening MRI was reported independently by two study radiologists (D.K., H.S.S., F.G., and S.P., with 6, 7, 8, and 16 years of mpMRI reporting experience, respectively) and categorised as ‘screen positive’, ‘screen negative’, or ‘non-diagnostic’ (Table 1), with a third radiologist in cases of disagreement. Radiologists were blinded to clinical details, including PSA. MRI acquisition parameters and further information on image reporting are included in Supplementary Methods.

**Table 1:** Screening MRI reporting criteria

Axial T2 (Likert)	Axial b2000 (Likert)	Screen outcome
Focal 4–5/5 or diffuse 3/5	Focal 4–5/5	Positive
Focal 4–5/5	Focal 1–2/5 or diffuse 3/5	Negative
Diffuse 3/5	1–2/5 or diffuse 3/5	Negative
1–2/5	ANY	Negative
ANY	1–2/5	Negative
Non-diagnostic (artefact)	1–2/5 or diffuse 3/5	Negative
1–2/5 or diffuse 3/5	Non-diagnostic (artefact)	Negative
4–5/5	Non-diagnostic (artefact)	Non-diagnostic (recall)
Non-diagnostic (artefact)	4–5/5	Non-diagnostic (recall)
Non-diagnostic (artefact)	Non-diagnostic (artefact)	Non-diagnostic (no recall)

Lesions scoring 3/5 on T2 with a focal area that is slightly more intense than background diffuse change (but not qualifying as 4/5) are encompassed under diffuse 3/5

Prostate-specific antigen density (PSAd) was calculated, and men with PSAd ≥ 0.12 ng/mL^2^ were categorised as ‘screen positive’, regardless of MRI result.

#### Post-screening pathway

A structured letter outlining the screening outcome was sent to the participant and their general practitioner (GP). GPs were advised to refer screen-positive men to secondary care via the two-week-wait suspected cancer pathway, following National Institute for Health and Care Excellence (NICE) guidelines. Screen-negative men exited the study.

Screen-positive participants were followed up to collect data from subsequent standard-of-care NHS investigations, including mpMRI and prostate biopsy if indicated, which were performed as per the local pathway. The decision to biopsy was made jointly between the clinical team and participant based on imaging findings, risk factors, and PSAd. Prostate biopsies were performed using a transperineal targeted approach with cognitive MRI/transrectal ultrasound fusion in 97% of cases; in 3%, additional systematic cores were also obtained. MpMRIs were reported by local clinical radiologists and included a Likert score. Subsequently, the mpMRIs were re-evaluated by a study radiologist (G.B.), blinded to biopsy outcome, using Prostate Imaging Reporting and Data System (PI-RADS) v2.1 criteria [18].

#### Two-year follow-up

The electronic healthcare records of screen-positive participants were examined 2 years following recruitment, and the outcomes of subsequent clinical review, MRI, and biopsy were documented. The screening MRIs and standard-of-care mpMRIs were retrospectively analysed by an experienced study radiologist (S.P.).

### Reference standard

The diagnostic accuracy of the screening MRI was evaluated using a composite reference standard (where available): 2-year electronic health record follow-up > biopsy outcome > mpMRI outcome. Screen-negative men were assumed to be the reference standard negative. Screen-positive men who were not referred to secondary care were excluded from the primary outcome. csPCa was defined as the presence of one biopsy core containing tissue with a Gleason score ≥ 3 + 4 [19].

Men with a positive screening MRI subsequently diagnosed with csPCa, including during the follow-up period, were defined as screening MRI-true-positive; men with a positive screening MRI but negative mpMRI (did not lead to biopsy) or negative biopsy, were defined as screening MRI-false-positive; men with a negative screening MRI subsequently diagnosed with csPCa, including during the follow-up period, were defined as screening MRI-false-negative; men with a negative screening MRI, negative mpMRI (did not lead to biopsy) or negative biopsy were defined as screening MRI-true-negative (Fig. S1).

### Statistical analysis

There were no formal sample size calculations as the study was designed as a feasibility study to assess the acceptance rate of a screening invitation and to determine the prevalence of MRI-defined suspicious lesions.

The primary outcome of our analysis was the PPV of the screening method for detecting csPCa, relative to a composite reference standard. Sensitivity, specificity, and NPV were not reported due to the absence of reference standard verification in screen-negative men; therefore, PPV was selected as the most appropriate diagnostic accuracy measure in this context. Summary statistics were used to describe the data. PPV was defined as the proportion of participants with a positive screening outcome who were found to have csPCa. Confidence intervals for the PPV were calculated using Wilson’s method [20]. Statistical analysis was performed by T.P. using Stata (v18.5, StataCorp).

## Results

### Participant characteristics

Of 2096 invited men, 309 were recruited with a median age of 62 years (interquartile range 56, 68) and median PSA of 1.2 ng/mL (interquartile range 0.7, 2.2). Recruitment flow chart and detailed baseline demographics have been previously published [17]. Summary characteristics of the participants are shown in Table 2. The prevalence of csPCa after initial assessment (i.e., not including 2-year follow-up) was 9.6% (29/303).

**Table 2:** Baseline and screening visit characteristics

Characteristic	Total participants
	*N* = 309
Age (years)	62 (56, 68)
Family history of PCa	
Absent	267 (86)
Present	41 (13)
Missing	1 (< 1)
Prostate volume (mL)^a^	29 (23, 38)
PSA (ng/mL)^b^	1.20 (0.74, 2.23)
PSAd (ng/mL^2^)^a^	0.04 (0.03, 0.06)

Data are *n* (percentage) or median (interquartile range)

*PSA* prostate-specific antigen, *PSAd* prostate-specific antigen density

^a^ *N* = 303

^b^ *N* = 306

### Screening outcome

**Fig. 1:**
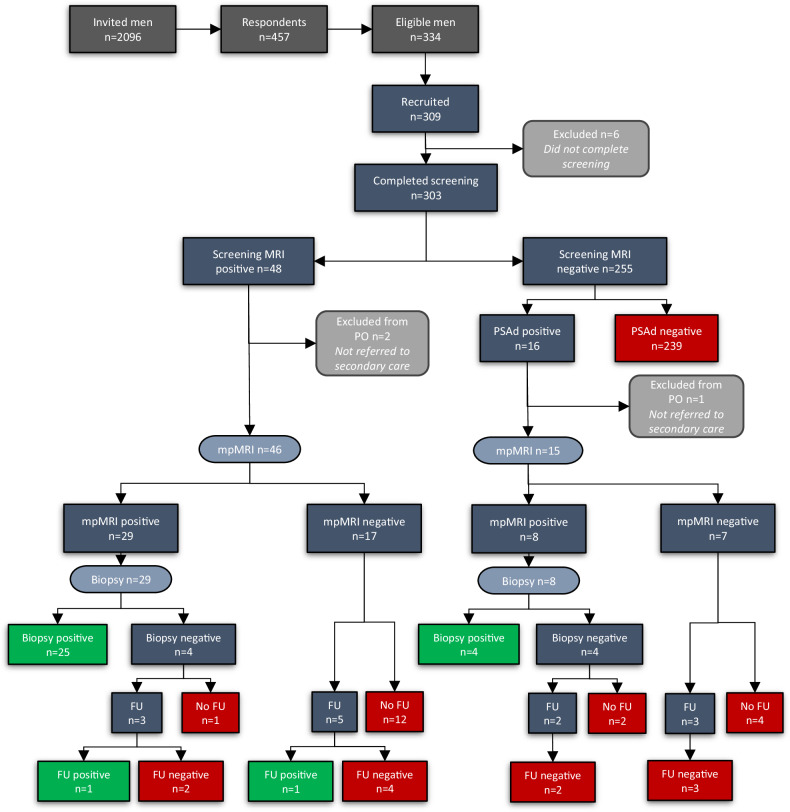
ReIMAGINE screening participant flowchart. PSAd ≥ 0.12 ng/mL^2^ was considered positive. Green boxes correspond to disease-positive, and red boxes correspond to disease-negative by the composite reference standard. PSAd, prostate-specific antigen density; mpMRI, multiparametric MRI; PO, primary outcome; FU, 2-year follow-up

Among 303 men who completed screening, 48 of 303 (16%) had a positive screening MRI, and 16 of 303 (5%) had a negative screening MRI but raised PSAd (≥ 0.12 ng/mL^2^). Thus, 64 of 303 (21%) men had a positive screening result (Fig. 1). Of the 48 men with a positive screening MRI, 11 (23%) also had raised PSAd.

The overall agreement between the readers for the screening MRI was 98% (95% CI: 95, 99) (Table S2). Of 303 scans, 24 (8%, 24/303) had non-diagnostic DWI due to artefact; in the absence of a suspicious lesion on T2WI, 23 of 24 (96%) had a screen-negative outcome by scoring criteria, with only one participant recalled for repeat imaging.

### Standard-of-care mpMRI outcomes

Of the men who completed screening (*N* = 303), 46 of 48 (96%) with a positive screening MRI and 15 of 16 (94%) with raised PSAd alone were referred for standard-of-care mpMRI. The remaining three participants were not referred due to clinical reasons or personal choice and are thus excluded from the subsequent analyses.

**Fig. 2:**
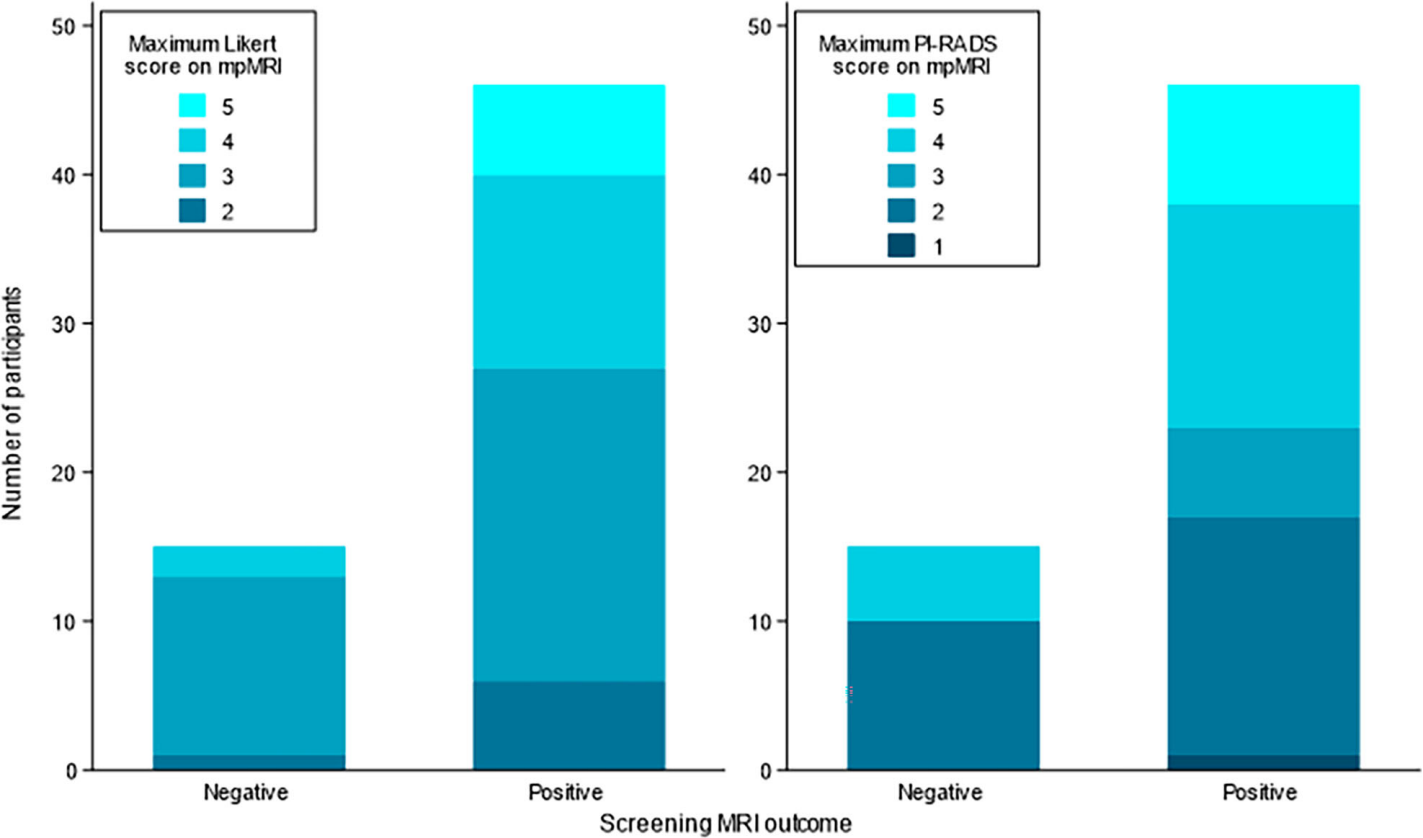
Maximum Likert and PI-RADS scores on standard-of-care mpMRI by screening MRI outcome. mpMRI, multiparametric MRI

Of 46 men referred with a positive screening MRI, 38 of 46 (83%) had at least one focal lesion (Likert/PI-RADS ≥ 3) described on mpMRI (Fig. S2) and in 36 of 38 (95%) men, this matched the lesion reported on the screening MRI. Of 15 men referred with a negative screening MRI but raised PSAd, 11 of 15 (73%) had at least one focal lesion described on mpMRI (Fig. S2). The Likert and PI-RADS scores on mpMRI are shown in Fig. 2.

Men with higher Likert and PI-RADS scores on mpMRI had higher rates of csPCa than men with lower Likert and PI-RADS scores (Table S3).

### Biopsy outcomes

Of 46 men referred with a positive screening MRI, 29 of 46 (63%) underwent biopsy; 25 of 46 (54%) had csPCa, 2 of 46 (4%) had non-significant PCa, and 2 of 46 (4%) had no cancer. Of 15 men referred with positive PSAd only, 8 of 15 (53%) underwent biopsy; 4 of 15 (27%) had csPCa, 1 of 15 (7%) had non-significant PCa, and 3 of 15 (20%) had no cancer.

Nine of 61 (15%) men referred to secondary care screened positive on both tests; 9 of 9 (100%) had csPCa, and 5 of 9 (56%) had predominant Gleason pattern 4 disease (Table 3).

**Table 3:** Biopsy outcomes in men referred to secondary care (*n* = 61) who screened positive on MRI only, positive on PSAd only and positive on both MRI and PSAd

Characteristic	Screening MRI positive and PSAd negative	Screening MRI negative and PSAd positive	Screening MRI positive and PSAd positive	All participants referred to secondary care
	*N* = 37	*N* = 15	*N* = 9	*N* = 61
PSAd (ng/mL^2^)	0.05 (0.04, 0.08)	0.15 (0.12, 0.26)	0.21 (0.16, 0.23)	0.10 (0.05, 0.15)
Maximum Gleason Score				
No cancer	2 (5)	3 (20)	0 (0)	5 (8)
3 + 3	2 (5)	1 (7)	0 (0)	3 (5)
3 + 4	15 (41)	3 (20)	4 (44)	22 (36)
4 + 3	1 (3)	1 (7)	1 (11)	3 (5)
4 + 5	0 (0)	0 (0)	4 (44)	4 (7)
No biopsy	17 (46)	7 (47)	0 (0)	24 (39)
Median MCCL (mm)	6 (3, 8)	6 (1, 7)	9 (8, 12)	7 (4, 9)

Data are *n* (percentage) or median (interquartile range)

*PSAd* prostate-specific antigen density, *MCCL* maximum cancer core length

### Two-year follow-up

On a 2-year follow-up of the electronic health records of the screen-positive men referred to secondary care (*N* = 61), two additional men (3%, 2/61) were diagnosed with csPCa, and both had positive screening MRI scans and normal PSAd. One man had a negative biopsy at the time of initial assessment (likely sampling error), and the other did not undergo biopsy. Table 4 summarises the composite reference standard outcomes; Table S4 provides participant-level outcomes for screen-positive men.

**Table 4:** Composite reference standard components (mpMRI, biopsy, and 2-year follow-up) stratified by overall reference standard outcome

Reference standard component	Overall reference standard		All participants
	Negative	Positive	
	*N* = 269	*N* = 31	*N* = 300^a^
Maximum Likert score on mpMRI			
2	7 (3)	0 (0)	7 (2)
3	21 (8)	12 (39)	33 (11)
4	2 (1)	13 (42)	15 (5)
5	0 (0)	6 (19)	6 (2)
No mpMRI	239 (89)	0 (0)	239 (80)
Maximum PI-RADS score on mpMRI			
1	1 (0)	0 (0)	1 (0)
2	24 (9)	2 (6)	26 (9)
3	2 (1)	4 (13)	6 (2)
4	3 (1)	17 (55)	20 (7)
5	0 (0)	8 (26)	8 (3)
No mpMRI	239 (89)	0 (0)	239 (80)
Maximum Gleason grade on initial biopsy			
No cancer	4 (1)	1 (3)^b^	5 (2)
3 + 3	3 (1)	0 (0)	3 (1)
3 + 4	–	22 (71)	22 (7)
4 + 3	–	3 (10)	3 (1)
4 + 5	–	4 (13)	4 (1)
No biopsy	262 (97)	1 (3)^b^	263 (88)
2-year follow-up outcome			
Negative	11 (4)	–	11 (4)
Positive	–	2 (6)	2 (1)
N/A	258 (96)	29 (94)	287 (96)

Data are *n* (percentage)

*mpMRI* multiparametric MRI, *N/A* not applicable, *PI-RADS* prostate imaging reporting and data system

^a^ Of the 303 participants who completed all screening tests, 3 men had positive screening tests but were not referred to secondary care and are excluded from the analysis

^b^ Includes the two screen-positive men who were only diagnosed with clinically significant PCa during the 2-year follow-up period. These figures show the outcomes following the initial standard-of-care assessment

### Diagnostic accuracy of the screening pathway

Of the 31 men diagnosed with csPCa overall (29 following initial referral and 2 during the follow-up period), 18 of 31 (58%) had normal PSAd, 15 of 31 (48%) had PSA < 3 ng/mL, and 2 of 31 (6%) had PSA < 1 ng/mL (Table 5). In comparison, 27 of 31 (87%) had a positive screening MRI.

**Table 5:** Screening test results (screening MRI, PSA, and PSAd) stratified by reference standard outcome

Screening test	Reference standard		All participants
	Negative	Positive	
	*N* = 269	*N* = 31	*N* = 300^a^
Screening MRI outcome			
Negative	250 (93)	4 (13)	254 (85)
Positive	19 (7)	27 (87)	46 (15)
PSAd outcome			
Negative	258 (96)	18 (58)	276 (92)
Positive	11 (4)	13 (42)	24 (8)
PSAd (ng/mL^2^)	0.04 (0.03, 0.05)	0.10 (0.06, 0.21)	0.04 (0.03, 0.06)
PSA ≥ 1 ng/mL	148 (55)	29 (94)	177 (59)
PSA ≥ 3 ng/mL	29 (11)	16 (52)	45 (15)

Data are *n* (percentage) or median (interquartile range)

*PSA* prostate-specific antigen, *PSAd* PSA density

^a^ Of the 303 participants who completed all screening tests, 3 men had positive screening tests but were not referred to secondary care and are excluded from the analysis

The PPV of the screening MRI alone was 54% (25/46, 95% CI: 40, 68%), rising to 86% (25/29, 95% CI: 69, 95%) when followed by mpMRI. The PPV of the complete pathway—a positive screening MRI and/or PSAd followed by mpMRI—was 78% (29/37, 95% CI: 63, 89%). Including the 2-year follow-up outcomes, the PPV of the screening MRI alone was 59% (27/46, 95% CI: 44, 72%). The Grade Group ≥ 2 (GG2+)/GG1 detection ratio was 12.5. Other benefit-to-harm ratios [21] are presented in Table S5.

#### Screening MRI-true-positive

**Fig. 3:**
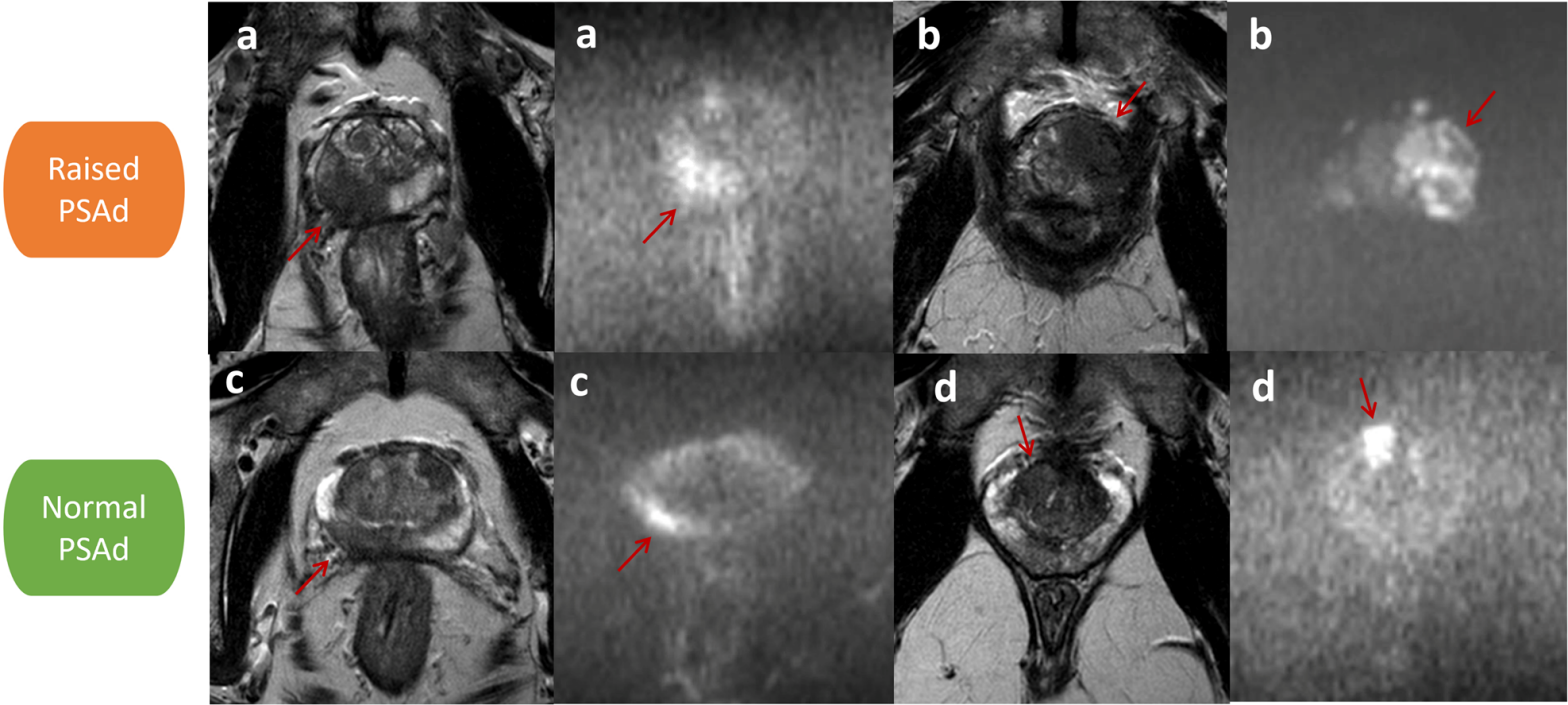
Examples of screening MRI-true-positive scans in men with raised (top) and normal (bottom) PSAd—paired T2-weighted imaging (T2WI) (left) and b2000 DWI (right) for each participant (**a**–**d**): **a** 72-year-old male with right peripheral zone (PZ) Gleason 4 + 5 tumour (arrow) with PSA 4.97 ng/mL and PSAd 0.18 ng/mL^2^; **b** 72-year-old male with left transitional zone (TZ)/PZ Gleason 4 + 5 tumour (arrow) with PSA 43.5 ng/mL and PSAd 0.75 ng/mL^2^; **c** 70-year-old male with right PZ Gleason 3 + 4 tumour (arrow) with PSA 1.84 ng/mL and PSAd 0.05 ng/mL^2^; and (**d**) 52-year-old male with right TZ Gleason 3 + 4 tumour (arrow) with PSA 2.42 ng/mL and PSAd 0.10 ng/mL^2^

After the 2-year follow-up period, 27 of 46 (59%) men were classified as screening MRI-true-positive (Fig. 3). Interestingly, two of these men were only diagnosed with csPCa during follow-up. In both men, the positive biopsy matched the lesion location reported on the initial screening MRI.

#### Screening MRI-false-positive

**Fig. 4:**
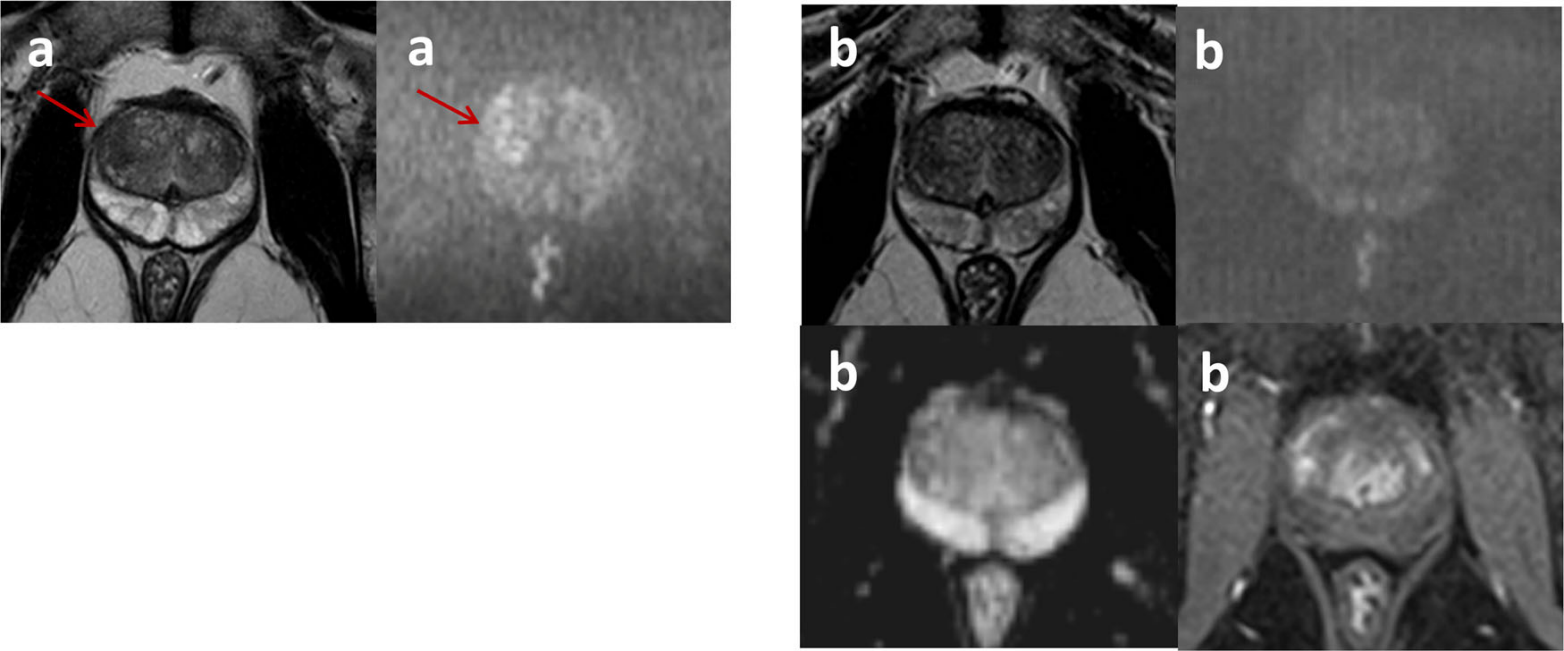
Example of a screening MRI-false-positive scan: (**a**) paired T2WI (left) and b2000 DWI (right) from the screening MRI of a 64-year-old male, which was reported as concordant positive due to a lesion in the right transition zone (arrow). There is a focal high signal on DWI but only a subtle change on T2WI. PSA was 0.81 ng/mL and PSAd 0.03 ng/mL^2^. **b** T2WI (top left), DWI (top right), ADC map (bottom left), and early dynamic contrast-enhanced images (bottom right) from the subsequent mpMRI show no focal abnormality. This was classified as a borderline overcall/limitation of the scoring system

Conversely, 19 of 46 (41%) men were classified as screening MRI-false-positive (Fig. 4). Retrospective imaging review showed 11 were borderline overcalls/a limitation of the scoring system (these men had equivocal changes on T2WI), 4 men had confounding pathology (for example, inflammation on biopsy), 2 had small lesions (possibility of biopsy sampling error) and 1 was related to technical factors (artefact affecting DWI) (Table S6). One participant was recalled following the second review of the mpMRI but declined follow-up. Further examples are included in Fig. S3.

#### Screening MRI-false-negative

**Fig. 5:**
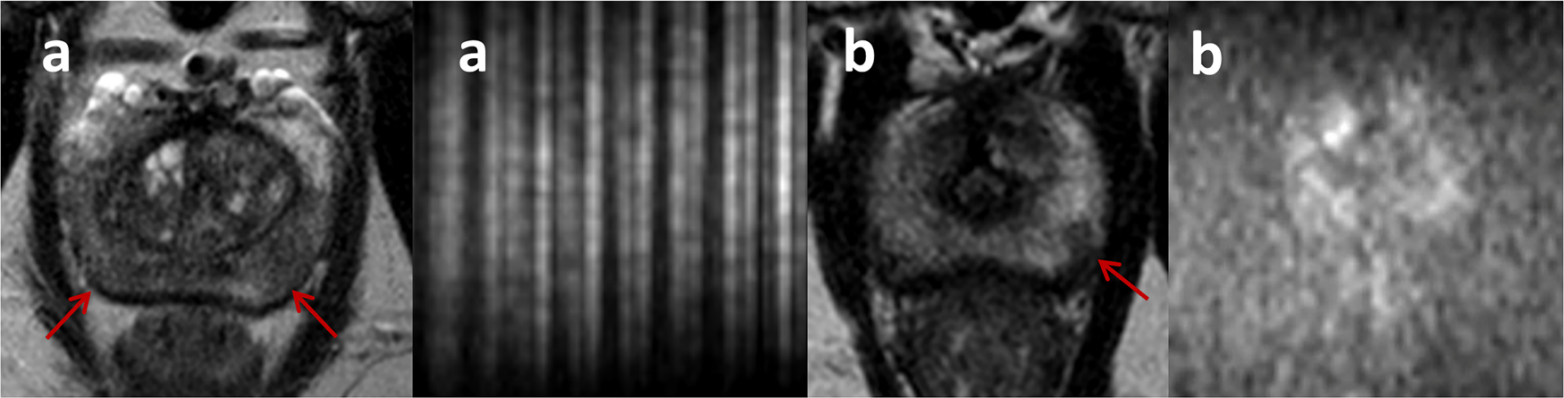
Examples of screening MRI-false-negative scans - paired T2WI (left) and b2000 DWI (right) for each participant (**a**, **b**): **a** 62-year-old male with PSA 6.42 ng/mL and PSAd 0.27 ng/mL^2^. Screening MRI was negative by ReIMAGINE criteria due to diffuse 3/5 change in the peripheral zones (PZ) and non-diagnostic DWI (due to coil malfunction). Biopsy showed right PZ Gleason 4 + 3 and left PZ Gleason 3 + 4 tumour (arrows); **b** 57-year-old male with PSA 9.22 ng/mL and PSAd 0.26 ng/mL^2^. The screening MRI was reported as negative. Retrospectively classified as a borderline undercall due to focal low signal on T2WI and equivocal changes on DWI in the left PZ at 4/5 o’clock, which was Gleason 3 + 4 on biopsy (arrow)

This could only be assessed in men with raised PSAd who were referred to secondary care and therefore have an available reference standard. Of these men, 4 of 15 (27%) were classified as screening MRI-false-negative (Fig. 5). Retrospective imaging review showed interpretation error in 2 men (borderline undercalls on screening MRI) and technical error in 2 men (coil malfunction that rendered the DWI non-diagnostic—in the absence of a suspicious (4–5/5) lesion on T2WI, these were classified as screen-negative by criteria (Table 1)).

No differences in histology were observed between men with csPCa who were screening MRI-false-negative and those who were screening MRI-true-positive; however, no men had truly MRI-negative (i.e., non-visible) disease.

## Discussion

The findings of ReIMAGINE support the role of abbreviated MRI in PCa screening. The screening MRI detected more csPCa than PSAd (≥ 0.12 ng/mL^2^), without increasing non-significant PCa diagnoses, achieving a PPV of 59%. Notably, 58% (18/31) of men with csPCa had normal PSAd, and 6% (2/31) had PSA < 1 ng/mL. Conversely, only 13% (4/31) of men with csPCa had negative screening MRIs, and half were of suboptimal quality. These findings suggest that MRI could add value to PCa screening independently of PSA testing.

Several studies comparing MRI and PSA-based screening strategies support these findings. IP1-PROSTAGRAM [13] found that bpMRI with a PI-RADS ≥ 4 threshold detected more csPCa than PSA ≥ 3 ng/mL, without increasing non-significant PCa diagnoses. Similarly, MVP [14] found that bpMRI (PI-RADS ≥ 4 threshold) outperformed PSA (≥ 2.6 ng/mL) in detecting csPCa. PROSA [15] demonstrated higher detection rates of csPCa using bpMRI alone (PI-RADS ≥ 3 threshold) compared to a combined pathway of bpMRI in men with PSA > 3 ng/mL.

The proportion of positive screening MRIs in ReIMAGINE (16%) is consistent with the 10–11% prevalence of PI-RADS ≥ 4 lesions in IP1-PROSTAGRAM [13] and MVP [14]. The higher prevalence of csPCa in ReIMAGINE (9.6%) compared to IP1-PROSTAGRAM [13] (4.2%) may reflect the older age inclusion criteria of ReIMAGINE.

The PPV observed in ReIMAGINE (59%) is higher than studies using Likert and PI-RADS scoring systems adapted for bpMRI, for example, the PPV of PI-RADS ≥ 4 pathways in IP1-PROSTAGRAM (29%, 11/38) [13] and MVP (46%, 11/24) [14]. This may be due to the different screening approach, where fewer sequences set a higher threshold for a ‘positive’ MRI, reducing false positives.

While the PPV is favourable compared to other cancer screening programmes—for example, the UK NHS breast cancer screening programme (PPV 13–17% [22])—many unnecessary biopsies were avoided by incorporating standard-of-care mpMRI prior to biopsy. However, a rate of 1 in 2 unnecessary secondary care referrals remains a challenge, and increasing the PPV of the screening approach is essential to avoid overburdening healthcare resources.

Half of the false-positive scans in ReIMAGINE had equivocal (3/5) T2WI changes with focal (4–5/5) changes on DWI. In some, the focal changes on DWI were not apparent on subsequent mpMRI, and in others, biopsy was not performed due to reassuring PSA and ADC maps. While further raising the threshold for a positive MRI (by requiring 4/5 scores on both T2WI and DWI) might improve PPV, this must be balanced against the risk of missing csPCa, as 15% of true-positive scans had this appearance.

In essence, ReIMAGINE screening was performed using a ‘stage-gated’ approach. The first stage was the abbreviated screening MRI, followed by standard-of-care mpMRI in screen-positive cases, with the PPV increasing to 86% after the second stage. However, a stage-gated approach could also be envisioned for a single MRI examination, where axial T2WI and high *b*-value images are reviewed first, with additional bpMRI sequences evaluated only if initial findings are positive. This approach in a single examination may also confer the benefit of increased PPV. Incorporating an additional *b*-value to allow ADC map derivation would slightly increase scan times, but the additional information would allow bpMRI-adapted PI-RADS scoring and artificial intelligence-assisted reconstruction software could be used to shorten acquisition times [23].

High-quality images are essential for MRI-based screening, particularly when using limited sequences, as illustrated by two false-negative cases with non-diagnostic DWI. Use of quality assessment tools, such as PI-QUAL v2 [24], and incorporation of adjunctive screening measures, like PSAd, may help to reduce the risk of false negatives.

The high inter-reader agreement (98%) of ReIMAGINE surpasses that previously reported for PI-RADS and Likert scoring systems [25–27], including the more comparable approach of detecting PI-RADS ≥ 3 lesions, which reached 93% agreement [25]. This high level of concordance likely reflects the expertise of readers at this single tertiary centre, as well as the use of a simplified binary (positive/negative) reporting system. These findings support the feasibility of a binary approach and challenge the need for double reading in similar settings.

The ReIMAGINE study has limitations. First, screen-negative participants (79%) did not undergo mpMRI or biopsy and therefore lack reference standard verification. To minimise potential verification bias in this context, sensitivity, specificity, and NPV were not reported. We hypothesise that the relatively high prevalence of csPCa supports the sensitivity of the screening method. Second, technical differences between the screening MRI (100% 3-T) and standard-of-care mpMRI (90% 1.5-T) may have influenced lesion appearance, particularly on high *b*-value sequences, which vary in diffusion-weighting on different field strength scanners. Finally, the single-centre design and use of expert readers may limit the generalisability of our findings. Prospective validation in multicentre settings with readers of varying experience levels is needed to confirm the broader applicability of our results. However, the successful implementation of prostate MRI screening may ultimately require specialist centres to ensure consistent reporting quality and standardisation of imaging protocols.

Abbreviated bpMRI shows promise in PCa screening and may improve cancer detection independently of PSA testing. However, challenges remain in optimising PPV, reducing unnecessary secondary care referrals, and managing economic and resource constraints. Our findings may inform the design of future multicentre PCa screening studies, such as the TRANSFORM trial [28], which will provide important evidence on the feasibility of MRI-based screening at a national level. Addressing these challenges could make MRI-based screening a viable and transformative approach for early PCa detection.

## Supplementary information

ELECTRONIC SUPPLEMENTARY MATERIAL

The ReIMAGINE Study Group

## Abbreviations

ADC: Apparent diffusion coefficient
bpMRI: Biparametric MRI
csPCa: Clinically significant prostate cancer
DWI: Diffusion-weighted imaging
mpMRI: Multiparametric MRI
NHS: National Health Service
NPV: Negative predictive value
PCa: Prostate cancer
PI-RADS: Prostate imaging reporting and data system
PPV: Positive predictive value
PSA: Prostate-specific antigen
PSAd: Prostate-specific antigen density
T2WI: T2-weighted imaging
