# Supplementary material for: Diagnostic accuracy of abbreviated biparametric MRI for prostate cancer screening: a prospective feasibility study (ReIMAGINE study)
Source: Eur Radiol. 2025 Aug 6;36(3):1959–70. doi: 10.1007/s00330-025-11837-1 (PMC12963090; doi:10.1007/s00330-025-11837-1)
Supplement: Supplementary file 1 — ELECTRONIC SUPPLEMENTARY MATERIAL [file 330_2025_11837_MOESM1_ESM.pdf]

Diagnostic accuracy of abbreviated biparametric MRI for prostate cancer screening: a prospective feasibility study (ReIMAGINE Study)

ELECTRONIC SUPPLEMENTARY MATERIAL

Supplemental Methods

Inclusion/Exclusion Criteria:

Inclusion criteria
1. Men aged 50–75 years.<br>2. No prior prostate cancer diagnosis or treatment.<br>3. Willing and able to provide written informed consent.

Exclusion criteria
1. Contraindication for MRI scanning (as assessed by the MRI safety questionnaire of the UCLH positron emission tomography/MRI department) which includes but is not limited to: intracranial aneurysm clips or other metallic objects; intraorbital metal fragments that have not been removed; pacemakers or other implanted cardiac rhythm management devices and non-MRI compatible heart valves; inner ear implants and history of claustrophobia.<br>2. Men who require assisted living, for example, care home living.<br>3. Dementia or other neurological conditions, meaning participant lacks the capacity to consent.

Screening MRI acquisition protocol:

Sequence parameters for the clinical and exploratory Luminal Water Imaging (LWF)[1]. All studies were performed with the same protocol on a single 3T scanner (Achieva, Philips Healthcare, Netherlands) using a 32-channel pelvic-phased array coil. The clinical sequences utilised an abbreviated biparametric MRI acquisition protocol. Total scan time was <20 minutes. The scan required no patient preparation.

Supplementary Table S1 – screening MRI acquisition parameters

**Parameter**	**T2 axial TSE**	**DWI b 2000s/mm²**	**LWF imaging**
**Repetition time (ms)**	5407	2000	8956
**Echo time (ms)**	100	78	31.25/31.25
**Flip angle (degree)**	90	90	90
**Orientation**	Axial	Axial	Axial
**Slice thickness**	3	5	4
**Matrix size**	300 x 290	168 x 169	300 x 290
**Field of view (mm)**	180	220	180
**Fat suppression**	No	SPIR	No
**Time for scan (min)**	05:13	03:40	05:13

DWI = diffusion-weighted imaging; LWF = Luminal Water Imaging

MRI reporting procedure:

The clinical sequences were reviewed independently by two study radiologists. The first reporter documented scan quality and an outcome of 'screen positive' or 'screen negative', along with prostate volume, presence, and location of lesions. The second reporter documented scan quality and a binary outcome of 'screen positive' or 'screen negative' but could include comments. If there was a disagreement between reporters, a third radiologist independently reported a binary outcome of 'screen positive' or 'screen negative'. Exploratory LWF images were not reported or used to determine screen status and will be retrospectively analysed.

Reference Standard:

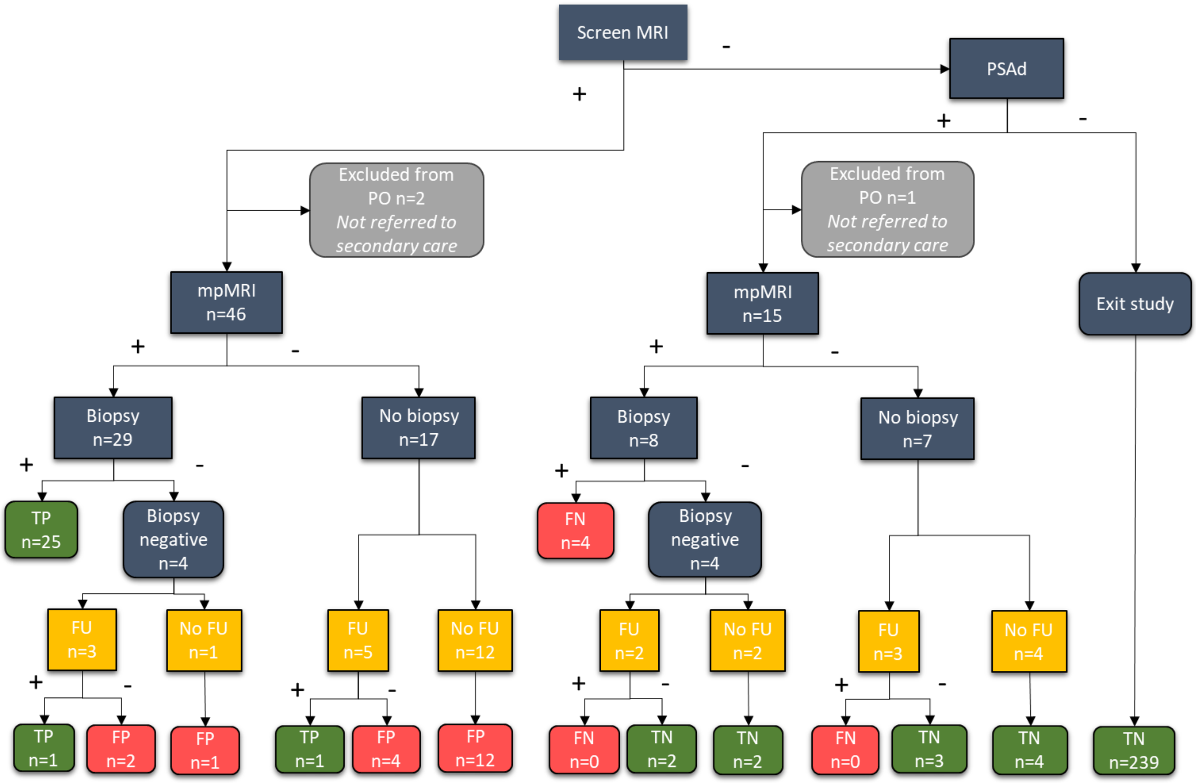

Supplementary Figure S1 - Flowchart demonstrating how diagnostic accuracy of the screening MRI was derived using reference standard outcomes.

PSAd = prostate-specific antigen density; PO = primary outcome; mpMRI = multiparametric MRI; FU = 2-year follow-up; TP = true positive; FP = false positive; TN = true negative; FN = false negative.

Supplemental Figures

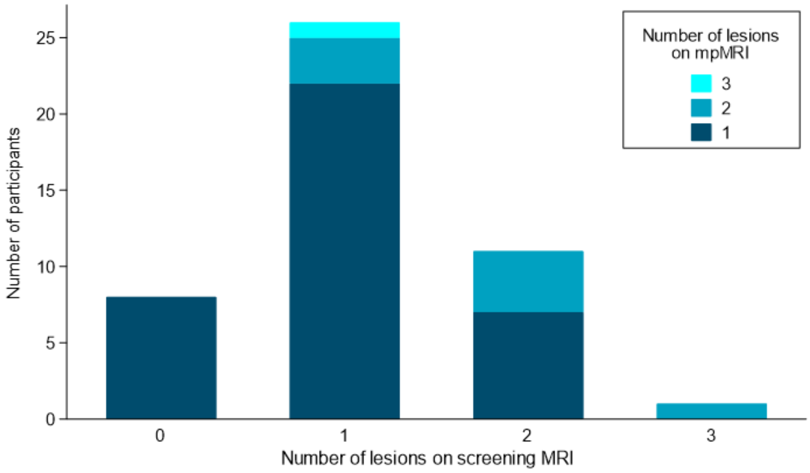

Supplementary Figure S2 – the number of lesions reported on the screening MRI compared to the number of lesions reported on subsequent multiparametric MRI (mpMRI) in men referred to secondary care (n=61).

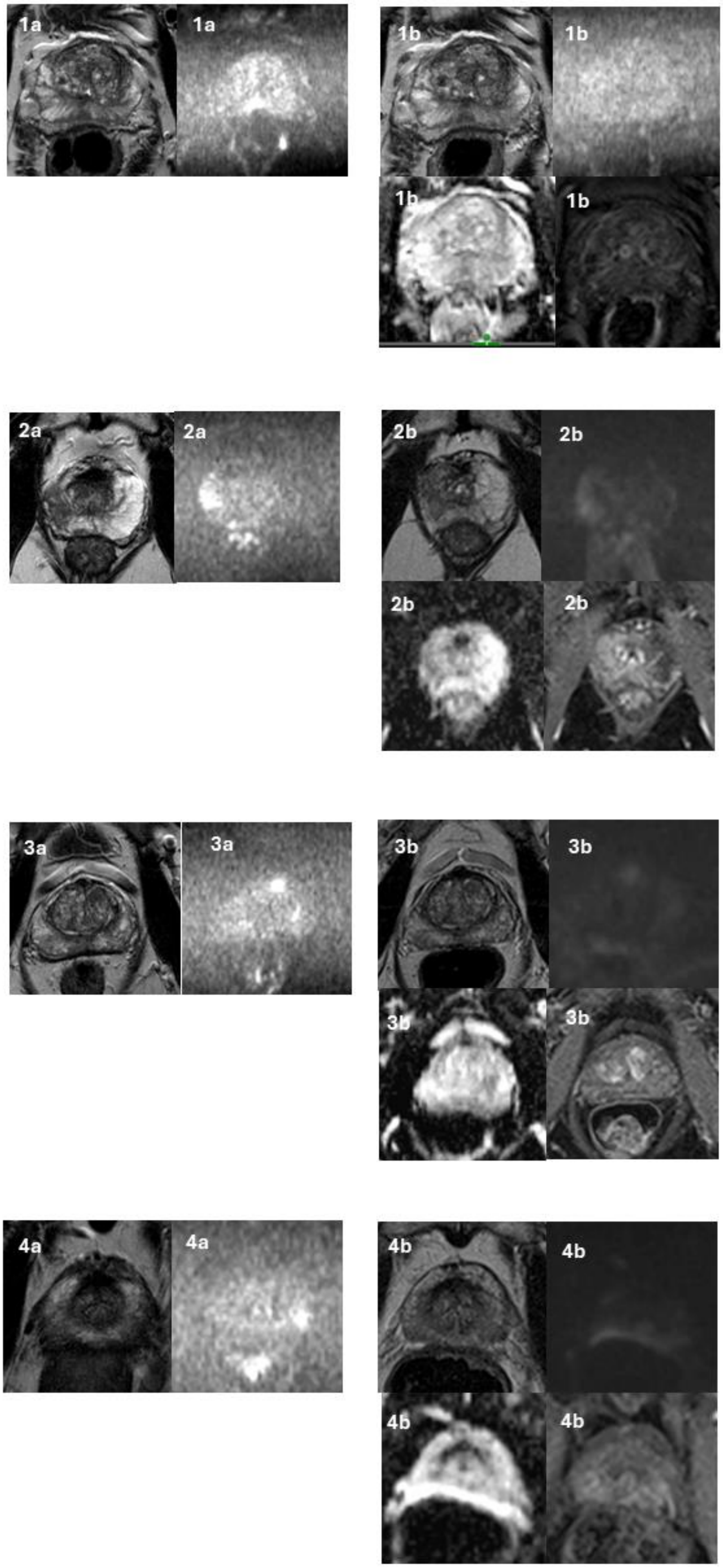
1a
1a
1b
1b
1b
1b
2a
2a
2b
2b
2b
2b
3a
3a
3b
3b
3b
3b
4a
4a
4b
4b
4b
4b

Supplementary Figure S3 – Further examples of screening MRI-false-positive men (1-4). For each participant, (a) paired T2-weighted (T2WI) (left) and b2,000 diffusion-weighted (DWI) (right) images from the screening MRI, and (b) T2WI (top left), DWI (top right), apparent diffusion coefficient map (bottom left) and early dynamic contrast-enhanced images (bottom right) from the subsequent multiparametric (mp)MRI, are shown.

- (1a) – 63-year-old male with positive screening MRI due to lesion at 6-7 o'clock in the peripheral zone (PZ). PSA was 2.15 ng/mL and PSAd 0.04 ng/mL^2; (1b) subsequent mpMRI shows no focal abnormality. Classified as false positive due to artefact on DWI.
- (2a and 2b) – 62-year-old male with positive screening MRI due to focal abnormality in the right PZ, also demonstrated on mpMRI. PSA was 1.16 ng/mL and PSAd 0.05 ng/mL^2. Biopsy of the lesion showed high grade prostatic intraepithelial neoplasia. Classified as false positive due to confounding pathology.
- (3a and 3b) – 68-year-old male with positive screening MRI due to focal abnormality in the left anterior transition zone (TZ), also demonstrated on mpMRI and graded 4/5. PSA was 1.50 ng/mL and PSAd 0.05 ng/mL^2. Biopsy of the lesion showed no cancer. Classified as false positive due to potential sampling error (difficult to access, small lesion).
- (4a) – 69-year-old male with positive screening MRI due to focal DWI abnormality at 4 o'clock in the left PZ with 3/5 change on T2WI. PSA was 0.63 ng/mL and PSAd 0.04 ng/mL^2 (4b) no focal abnormality seen on mpMRI (although DWI affected by artefact). Classified as false positive due to borderline overcall/limitation of scoring system.

Supplemental Tables

Supplementary Table S2 – Inter-reader agreement on screening MRI outcome.

**Inter-reader agreement**	**Total Participants N=303**
Reader 1 positive & Reader 2 positive	45
Reader 1 positive & Reader 2 negative	2
Reader 1 negative & Reader 2 negative	251
Reader 1 negative & Reader 2 positive	5
Negative-specific agreement	99 (97, 99)
Positive-specific agreement	93 (89, 95)
Overall agreement	98 (95, 99)
Data are n or % (Wilson's 95% confidence interval)	

Supplementary Table S3 – Maximum multiparametric MRI Likert/PI-RADS score and per-patient biopsy outcomes in men referred to secondary care (n=61).

**Biopsy outcome**	**Maximum Likert Score**					**Maximum PI-RADS Score**				
	**1**	**2**	**3**	**4**	**5**	**1**	**2**	**3**	**4**	**5**
	N=0	N=7	N=33	N=15	N=6	N=1	N=26	N=6	N=20	N=8
No cancer	0 (0)	0 (0)	4 (12)	1 (7)	0 (0)	0 (0)	3 (12)	1 (17)	1 (5)	0 (0)
nsPCa	0 (0)	0 (0)	2 (6)	1 (7)	0 (0)	0 (0)	2 (8)	0 (0)	1 (5)	0 (0)
csPCa	0 (0)	0 (0)	10 (30)	13 (87)	6 (100)	0 (0)	1 (4)	3 (50)	17 (85)	8 (100)
No biopsy	0 (0)	7 (100)	17 (52)	0 (0)	0 (0)	1 (100)	20 (77)	2 (33)	1 (5)	0 (0)

Data are n or n (%)
csPCa = clinically significant prostate cancer; nsPCa = non-significant prostate cancer

Supplementary Table S4 – Reference standard outcomes for each participant referred to secondary care (n=61). Two-year follow-up and reference standard assessments are based on electronic healthcare record review and retrospective image analysis.

Screening MRI outcome	Screening MRI scan quality	PSAd (ng/mL^2)	Maximum Likert score	Maximum Gleason grade	MCCL	2-year follow-up outcome	2-year follow-up comments	Reference standard	Reference standard comments
Negative	Diagnostic	0.12	2					Negative	
Negative	Diagnostic	0.12	3	3+3	1	Negative	Repeat biopsy 3+3	Negative	
Negative	Diagnostic	0.12	3			Negative	Stable MRI	Negative	
Negative	Diagnostic	0.12	3			Negative	Stable MRI	Negative	
Negative	Diagnostic	0.12	3					Negative	
Negative	Non-diagnostic	0.13	4	3+4	1			Positive	DWI non-diagnostic (technical error with coil)
Negative	Diagnostic	0.14	3			Negative	Stable MRI and no cancer on subsequent biopsy	Negative	
Negative	Diagnostic	0.15	3	No cancer				Negative	
Negative	Diagnostic	0.16	3					Negative	
Negative	Diagnostic	0.23	3	No cancer		Negative	Stable MRI	Negative	
Negative	Diagnostic	0.23	3	3+4	12			Positive	Borderline undercall
Negative	Diagnostic	0.26	3					Negative	
Negative	Diagnostic	0.26	4	3+4	7			Positive	Borderline undercall
Negative	Non-diagnostic	0.27	3	4+3	6			Positive	DWI non-diagnostic (technical error with coil)
Negative	Diagnostic	0.38	3	No cancer				Negative	
Positive	Diagnostic	0.02	3					Negative	Borderline overcall/limitation of scoring system

Positive	Diagnostic	0.02	4	3+4	12			Positive	
Positive	Diagnostic	0.03	2					Negative	Borderline overcall/limitation of scoring system
Positive	Diagnostic	0.03	2					Negative	Borderline overcall
Positive	Diagnostic	0.03	3	3+4	3			Positive	
Positive	Diagnostic	0.03	3	3+4	5			Positive	
Positive	Diagnostic	0.03	3					Negative	Borderline overcall
Positive	Diagnostic	0.04	2					Negative	DWI artefact
Positive	Diagnostic	0.04	3			Negative	Changes resolved on repeat MRI	Negative	Confounding pathology (inflammation)
Positive	Diagnostic	0.04	3			Negative	Stable MRI	Negative	Confounding pathology (adenoma)
Positive	Diagnostic	0.04	3			Negative	Stable MRI	Negative	Borderline overcall/limitation of scoring system
Positive	Diagnostic	0.04	3					Negative	Borderline overcall/limitation of scoring system
Positive	Diagnostic	0.04	3					Negative	Declined recommended follow-up
Positive	Diagnostic	0.04	4	3+4	3			Positive	
Positive	Diagnostic	0.04	4	3+4	8			Positive	
Positive	Diagnostic	0.05	2					Negative	Borderline overcall/limitation of scoring system
Positive	Diagnostic	0.05	2					Negative	Borderline overcall/limitation of scoring system
Positive	Diagnostic	0.05	3	3+3		Negative	Stable MRI	Negative	Confounding pathology (PIN)

Positive	Diagnostic	0.05	3					Negative	Borderline overcall/limitation of scoring system
Positive	Diagnostic	0.05	4	No cancer				Negative	Possible sampling error
Positive	Diagnostic	0.05	4	3+4	8			Positive	
Positive	Diagnostic	0.05	5	3+4	10			Positive	
Positive	Diagnostic	0.06	2					Negative	Borderline overcall/limitation of scoring system
Positive	Diagnostic	0.06	3	3+4	7			Positive	
Positive	Diagnostic	0.06	5	3+4	8			Positive	
Positive	Diagnostic	0.08	3	4+3	3			Positive	
Positive	Diagnostic	0.08	3					Negative	Borderline overcall/limitation of scoring system
Positive	Diagnostic	0.08	4	3+4	5			Positive	
Positive	Diagnostic	0.09	4	3+3	2	Negative	MRI stable	Negative	Possible sampling error
Positive	Diagnostic	0.09	5	3+4	4			Positive	
Positive	Non-diagnostic	0.10	3	3+4	2			Positive	
Positive	Diagnostic	0.10	3			Negative	PSA decreasing	Negative	Confounding pathology (inflammation)
Positive	Diagnostic	0.10	3			Positive	Stable MRI, subsequent biopsy 3+4	Positive	
Positive	Diagnostic	0.10	4	3+4	6			Positive	
Positive	Diagnostic	0.10	4	3+4	10			Positive	
Positive	Diagnostic	0.10	5	3+4	6			Positive	

Positive	Diagnostic	0.11	3	No cancer		Positive	Progression on MRI, repeat biopsy 3+4	Positive	
Positive	Diagnostic	0.13	3	3+4				Positive	
Positive	Diagnostic	0.15	4	3+4	9			Positive	
Positive	Diagnostic	0.16	3	3+4	9			Positive	
Positive	Diagnostic	0.18	5	4+5	12			Positive	
Positive	Diagnostic	0.21	3	4+3				Positive	
Positive	Diagnostic	0.21	4	3+4	6			Positive	
Positive	Diagnostic	0.23	4	4+5	9			Positive	
Positive	Diagnostic	0.33	4	4+5	12			Positive	
Positive	Diagnostic	0.75	5	4+5	8			Positive	

PSAd = prostate-specific antigen density; MCCL = maximum cancer core length; DWI = diffusion-weighted imaging; PIN = prostatic intraepithelial neoplasia

Supplementary Table S5 – benefit-to-harm ratios[2] of the ReIMAGINE screening pathway. The *GG2+/GG1 ratio* reflects the selectivity of the screening strategy for detecting clinically significant cancers (GG2+), while minimising the detection of insignificant cancers (GG1). The *GG2+/GG1+GG0 ratio* considers the overall accuracy of the screening strategy, including the number of unproductive biopsies (GG0, no cancer). The *GGx/GG0 ratio* (i.e. avoided biopsies/unproductive biopsies) reflects the balance of MRI negative screens avoiding biopsy to unproductive biopsies in MRI positive screens.

**Outcome**	**Total Participants N=303**
Screening MRI negative men (i.e. avoided biopsy)	255
Screening MRI positive men	48
Biopsy outcome in screening MRI positive men	
No biopsy	19 (40)
GG0	2 (4)
GG1	2 (4)
GG2+	25 (52)
Harm-to-benefit ratios	
GG2+/GG1	12.5
GG2+/GG1+GG0	6.3
GGx/GG0	128.0

Data are n or n (%)
GG = grade group; GG2+ = clinically significant cancer; GG1 = clinically insignificant cancer; GG0 = no cancer; GGx = avoided biopsies; ‘avoided biopsy’ = screening MRI negative men.

Supplementary Table S6 - Reasons for false positive findings on the screening MRI

**Classification**	**False Positive Participants N=19**
Borderline overcall/limitation of scoring system	11
Confounding pathology	4
Small lesion	2
Technical factor (artefact)	1
Recalled but declined follow up	1

References

1. Devine W, Giganti F, Johnston EW, et al (2019) Simplified Luminal Water Imaging for the Detection of Prostate Cancer From Multiecho T2 MR Images. Journal of Magnetic Resonance Imaging 50:. https://doi.org/10.1002/jmri.26608

2. Schoots IG, Haider MA, Punwani S, Padhani AR (2025) MRI in Prostate Cancer Screening: A Review and Recommendations, From the AJR Special Series on Screening. AJR Am J Roentgenol. https://doi.org/10.2214/AJR.24.32588
