# Supplementary material for: Diagnostic accuracy of abbreviated biparametric MRI for prostate cancer screening: a prospective feasibility study (ReIMAGINE study)
Source: Eur Radiol. 2025 Aug 6;36(3):1959–70. doi: 10.1007/s00330-025-11837-1 (PMC12963090; doi:10.1007/s00330-025-11837-1)
Supplement: Supplementary file 2 — The ReIMAGINE Study Group [file 330_2025_11837_MOESM2_ESM.docx]

The authors would like thank members of The ReIMAGINE Study Group: Eric Aboagye, Hashim Ahmed, Fatima Akbar, Gerhardt Attard, Teresita Beeston, Charlotte Bevan, Chris Brew-Graves, Mrishta Brizmohun, Paul Boutros, Giorgio Brembilla, Louise Brown, Joey Clemente, Rosie Clow, Ton Coolen, Ged Corbett, Caroline Dive, Eytan Domany, Mark Emberton, Andrew Feber, Elena Frangou, Alex Freeman, Francesco Giganti, Miriam Gonclaves, Fiona Gong, Saran Green, Joanna Hadley, Ashling Henderson, Elizabeth Isaac, Richard Kaplan, Douglas Kopcke, Stefano Lise, Annabel Kunzemann Martinez, Teresa Marsden, Malcolm Mason, Neil McCartan, Caroline Moore, Charlotte Moss, Kinnari Naik, Anwar Padhani, Peter Parker, Chris Parker, Shonit Punwani, Nahian Rahman, Francesca Rawlins, Manuel Rodriguez-Justo, Mark Rowley, Aida Santa-Olalla, Harbir Sidhu, Pirruntha Sivaharan, Katerina Soteriou, Andrew Stubbs, Tom Syer, Suparna Thakali, Steve Tuck, Mieke Van Hemelrijck, Anna Wingate, Daniel Wetterskog, Hayley Whitaker, Savahnna Wolfe.
